# A Numerical Model for Understanding the Development of Adhesion during Drying of Cellulose Model Surfaces

**DOI:** 10.3390/ma16041327

**Published:** 2023-02-04

**Authors:** Magdalena Kaplan, Sören Östlund

**Affiliations:** Solid Mechanics, Department of Engineering Mechanics, KTH Royal Institute of Technology, 100 44 Stockholm, Sweden

**Keywords:** adhesion modelling, cohesive interactions, cellulose model surfaces, fibre–fibre joints

## Abstract

Adhesion is crucial for the development of mechanical properties in fibre-network materials, such as paper or other cellulose fibre biocomposites. The stress transfer within the network is possible through the fibre–fibre joints, which develop their strength during drying. Model surfaces are useful for studying the adhesive strength of joints by excluding other parameters influencing global performance, such as geometry, fibre fibrillation, or surface roughness. Here, a numerical model describes the development of adhesion between a cellulose bead and a rigid surface using an axisymmetric formulation, including moisture diffusion, hygroexpansion, and cohesive surfaces. It is useful for studying the development of stresses during drying. A calibration of model parameters against previously published contact and geometry measurements shows that the model can replicate the observed behaviour. A parameter study shows the influence of cohesive and material parameters on the contact area. The developed model opens possibilities for further studies on model surfaces, with quantification of the adhesion during pull-off measurements.

## 1. Introduction

Fibre network materials are a composite type with two constituting parts: the individual fibres and the interactions between them. When subjecting a network material to loading, forces transfer between the fibres through the points of contact, or fibre joints. The strength of the fibre joints thus becomes decisive for the strength of the composite.

Various kinds of fibres (most commonly cellulose-based) can make up these materials. Amongst these, many consist of paper pulp fibres. In paper making, pulp fibres are wetted to form a slurry, then pressed and dried to form the desired product or material. The removal of moisture changes the properties of individual fibres, but most importantly the network joints develop when the slurry transforms into a cohesive material. This means that the joints in the network develop their strength during drying [1].

To achieve the desired properties of fibre biocomposites, understanding the development of the joints is necessary. One course of action is manipulating pulp fibres and studying the properties of complete networks [2]. Fibre network modelling is another useful tool, where specific properties of fibres or joints can be varied and evaluated at a global scale [3,4,5]. Testing and evaluating laboratory-made fibre joints is also an option used in previous studies [6,7]. However, it may be difficult to isolate the influence of joint strength in all those cases, as many other parameters can influence performance; for example, the number of fibre contacts, fibrillation or surface roughness, and strength of individual fibres can all impact macroscopic behaviour [8,9]. Cellulose model surfaces have previously been useful in studying the effect of specific modifications on adhesive properties [10,11]. The present study uses model surfaces in the shape of solid homogenous beads, described and characterised in previous publications [12,13,14,15,16].

Several analytic models have been developed to describe adhesive behaviour between two surfaces. The JKR- [17] and DMT-models [18] are among the most known and are based on Hertz’s [19] theory of deformation between elastic bodies. The JKR-model accounts for large and soft bodies, where the external loading is small, and attractive forces caused by the surface energy enlarge the contact area. On the other hand, the DMT-model applies to stiff and small bodies, where the contact area expands due to van der Waals forces, but the contribution to the force needed to separate the surfaces vanishes. Maugis [20] later developed a model bridging the two extreme behaviours by assuming a constant stress at the edge of the contact zone and determining the stress concentration factor.

More recent studies cover the process of contact formation and how boundary conditions influence adhesion. Thouless and Jensen [21] conclude that residual stresses introduced during contact formation affect the loading mode and have significant effects on the adhesive properties. Olsson and Larsson [22] developed a model for adhesion between stiff, spherical particles subjected to compressive loading, based partly on Brinell indentation, and verified it using numerical simulations. Peng et al. [23] showed that the measured adhesion strength also depends on the geometry and size of the contacting objects. Other studies have also recently reported on other factors affecting adhesion, such as shear stresses, electric or magnetic potential, and rate dependency [24,25,26].

The drying process of single droplets has been widely investigated in other fields, such as pharmaceuticals and food technology [27]. Perdana et al. analysed the changes in geometry and enzyme activity for a single droplet, experimentally and numerically using an effective diffusion approach [28,29]. In contrast, the drying of cellulose beads is not studied as extensively and previous studies, especially, lack a closer investigation of the development of adhesive properties during drying. A suitable tool for studying the drying process is numerical modelling. Publications in the literature use numerical models as a way of describing and investigating the adhesive properties of fibre networks [3]. In particular, cohesive zone models, as pioneered by Dugdale [30] and Barenblatt [31] are useful for describing adhesive behaviour, where the contact stress development defines the strength of the joint. Lin et al. [32] experimentally and numerically characterised the moisture-dependent constitutive behaviour of paper fibres and implemented degradation of the cohesive joints similar to hydrogen embrittlement of steel. Previous studies have also investigated the dependence of both moisture and temperature on adhesive properties for other materials [33,34,35]. This work uses a cohesive zone model to simulate the behaviour of a cellulose bead during the drying process in a finite element (FE) model, quantifying the moisture-dependent adhesive properties.

This paper presents a numerical model replicating the geometry development of the cellulose bead model surface during drying. It is organised as follows: first, the method section presents the modelling objective and the numerical methods, including the geometry of the FE model, the assumptions made of the material description, and boundary conditions. An explanation of the cohesive interaction definition used for the adhesive description follows. The method section further describes the parameter fitting procedure and the parameter study. Finally, the results section presents the determined material and cohesive properties dependent on moisture. Analysis and discussion of the significance of the work follow, and the paper concludes by stating the future applications and further developments of the model. The novelty of the presented work lies in the determination of stresses developed during drying and how these affect the development and degradation of the joint strength. It is also possible to study the effect of individual material parameters and it opens the possibility to study complex phenomena in fibre-joint formation by understanding which parameters cause them, without including the additional difficulties resulting from the complex geometries of individual fibre–fibre joints. The understanding of the mechanics during drying complements the role of surface chemistry and is a step on the way towards fully understanding fibre–fibre joint formation.

## 2. Materials and Methods

The numerical investigation, including model development and pre- and postprocessing, used the FE-software ABAQUS 2019 [36].

### 2.1. Modelling Objective

One method of studying adhesion between two entities is the use of model surfaces. They allow for isolating and quantifying adhesive properties alone, without the influence of other factors on global performance. For example, fibre network materials owe their properties partly to the adhesion between individual fibre–fibre joints, but also to individual fibre properties, surface fibrillation, friction, entanglements, geometry, etc. Using an idealised geometry with a smooth surface makes it possible to examine only the adhesion properties. Adhesion testing measures the force required to separate contacting surfaces, in this case the bead and an underlying flat surface. The measured force depends on the process leading up to the contact formation; when the bead dries, stresses will develop, and the observed adhesive properties will change.

The modelling object used in this study was a nanometre-smooth, water-swollen cellulose bead made from regenerated cellulose. The resulting material is amorphous and isotropic [12]. The bead was set to dry on a hard surface covered with Kapton tape, and the geometries of the bead and contact zone were measured over the course of drying by Li et al. [15]. Section 2.3 explains the experimental data used further.

### 2.2. Numerical Model

**Figure 1:**
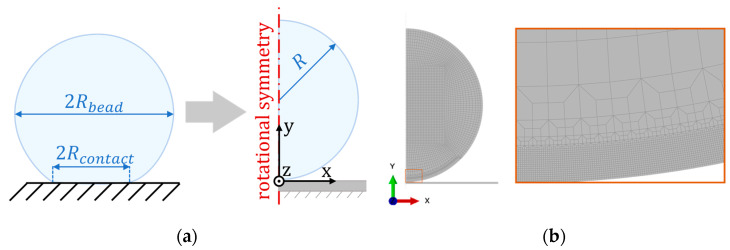
Model symmetries (**a**) and final FE-model with mesh used, including a close-up of transition elements (**b**).

The developed model represents the drying process, including moisture diffusion and shrinkage. The model uses a two-dimensional axisymmetric geometry, assuming rotational symmetry because of the spherical shape of the bead. Figure 1a shows the geometry of the bead and underlying rigid surface; the radius of the bead R corresponds to the wet bead radius, which is set to 0.63 mm according to experimental measurements from Li et al. [15]. The bead model was discretised using 4-noded quadrilateral elements, which is suitable for a contact analysis. The element size at the contact zone was small, with a dimension of about R/250. A transition zone allowed the element size far from the contact zone to be about ten times larger. Figure 1b shows the mesh and close-up of the transition region. The class of elements used depends on the analysis type, as explained further in Section 2.2.1. Comparison of a simple test case to the Hertz contact model [19] verified the validity of the mesh discretisation. A simplified model with linear elastic material on both bead and surface, and an applied compressive force yielded results consistent with the analytic solution.

A rotational symmetry line with a zero-displacement constraint in the x-direction prevented both the bead and surface from moving horizontally. Additionally, a fixed constraint at the bottom edge of the underlying surface inhibited it from movement in all degrees of freedom.

#### 2.2.1. Boundary Conditions and Analysis Steps

A sequential procedure allowed for solving the moisture-expansion problem in two separate analyses. First, by solving the moisture field in a coupled stress-diffusion problem, where a moisture boundary condition placed on the external edge of the bead allowed the moisture to diffuse into the bulk of the bead. The diffusion problem used 4-node axisymmetric hybrid thermally coupled quadrilateral, bilinear displacement and temperature, constant pressure elements (CAX4HT) [36].

**Figure 2:**
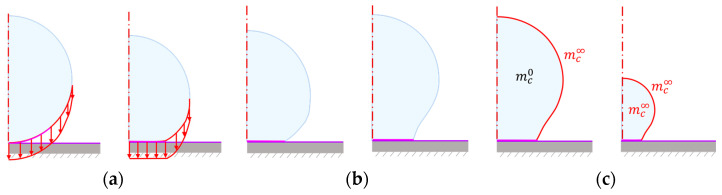
The three steps of the stress-shrinkage analysis with illustrated boundary conditions: (**a**) compaction, (**b**) unloading, and (**c**) shrinkage.

The second analysis, a stress procedure with cohesive interactions, consisted of three steps: compaction, loading, and drying, as summarised in Figure 2. The first two are preparation steps to get the correct geometry before the drying begins. In experiments, when placing the bead on the surface, it immediately deforms due to capillary forces pulling it towards the surface. A vertical pressure on the lower edge of the bead replaced the capillary forces, deforming it against the surface (compaction). After releasing the pressure (unloading), the cohesive interaction prevents the bead from separating from the surface and the initial deformation emerges.

The drying step used the resulting moisture field from the stress-diffusion analysis as a boundary condition. As the moisture changes, it causes hygroscopic strains to develop, and eventually, the bead starts to shrink. The ambient moisture condition was replaced by a boundary condition at the initially free edge of the bead; the rate of applied moisture was fit to give the same radius evolution as the experimental data, with the final moisture value corresponding to the ambient moisture. The free edge will extend when the contact area begins to fail making the boundary condition application a simplification. The elements used in the stress analysis were 4-node bilinear elements, hybrid with constant pressure (CAX4H) [36].

#### 2.2.2. Material Models

Li et al. [14] measured the elastic modulus of the cellulose beads using atomic force microscopy (AFM). The resulting stiffness was 0.45 MPa in the wet state and 12 MPa in the dry state. These measurements are the basis of the constitutive model used in this study.

Elastic–plastic material models describe the behaviour of many cellulose materials well. Marin et al. showed that a linear variation in moisture ratio agreed with the constitutive behaviour of paperboard [37]. However, this concerns low moisture levels: with relative humidity in the range of 20–90%, resulting in moisture ratios of less than 0.2. In the wet state, paper/cellulose holds much greater amounts of water, and the mechanical properties are drastically different. Like the wet surface of a pulp fibre [38], the cellulose bead is a hydrogel in the wet state [39]. The chosen material model must thus account for a soft, gel-like behaviour in the wet state and elastic in the dry state. A hyperelastic model captures the soft behaviour in the wet state and allows fitting of the input parameters as moisture dependent functions to account for the behaviour in the dry state. An isotropic Mooney-Rivlin [40,41] model with three parameters achieves this using the strain energy potential function

(1) $$W=C_{10}\left({\bar{I}}_{1}-3\right)+C_{01}\left({\bar{I}}_{2}-3\right)+\frac{1}{D_{1}}{\left(J^{el}-1\right)}^{2},$$

where ${\bar{I}}_{1}$ and ${\bar{I}}_{2}$ are the first and second deviatoric strain invariants, and $J^{el}$ is the elastic volume ratio. Linear elastic materials parameters approximated the coefficients $C_{10}$ and $C_{01}$, and $D_{1}$ using the relations

(2) $$C_{10}=\frac{G}{2\left(1+\frac{C_{01}}{C_{10}}\right)},$$

and

(3) $$D_{1}=2/K.$$

Here, G is the shear modulus defined by the elastic modulus of the material E and Poisson’s ratio ν as:

(4) $$G=\frac{E}{2\left(1+\nu \right)},$$

and K is the bulk modulus, also defined by E and ν as

(5) $$K=\frac{E}{3\left(1-2\nu \right)}.$$

**Figure 3:**
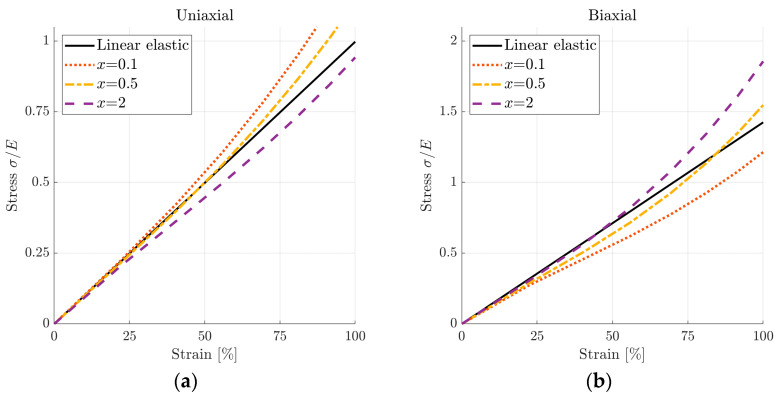
Comparison of a hyperelastic material model to linear-elastic for a simple one-element case in (**a**) uniaxial loading and (**b**) biaxial loading.

The hyperelastic model aims at mimicking the linear-elastic material behaviour as well as possible. Rewriting Equation (2) with the ratio

(6) $$x=C_{01}/C_{10},$$

allows for a parameter investigation. The linear-elastic and hyperelastic models were compared using a simple model with one two-dimensional quadrilateral element. Applying uniaxial and biaxial loading to the element allowed for a comparison of the resulting stress-strain plots, as shown in Figure 3. The value of x = 0.5 resulted in the best agreement with the linear-elastic curve. This ratio gave the hyperelastic coefficients throughout the simulations. Poisson’s ratio was assumed to be 0.3.

The values for the elastic modulus E measured by Li et al. [14] determined the moisture dependence of the hyperelastic parameters. The moisture ratio $m_{r}$ depends on the mass of water $m_{water}$ and dry content $m_{dry}$ as

(7) $$m_{r}=\frac{m_{water}}{m_{dry}}.$$

The moisture content $m_{c}$ has a non-linear relationship with the moisture ratio,

(8) $$m_{c}=\frac{m_{water}}{m_{water}+m_{dry}}=\frac{m_{r}}{1+m_{r}}.$$

Assuming that the relationship between E and $m_{r}$ is linear, a non-linear relationship between E and $m_{c}$ emerges. The parameter study evaluates this assumption, comparing it to another dependency, see Section 2.4. Moisture transport within the bead followed the moisture diffusion equation, describing the development of $m_{c}$ over time as

(9) $$\frac{\partial m_{c}}{\partial t}=\alpha \nabla^{2}m_{c},$$

where α is the diffusion coefficient. The hygroscopic strain $\varepsilon_{h}$ depends linearly on $m_{c}$ as

(10) $$\varepsilon_{h}=\beta \Delta m_{c},$$

where β is the expansion coefficient. The assumed moisture content of the bead was $m_{c}^{0}$ = 0.9 at the onset of drying and $m_{c}^{\infty }\;$= 0.1 after drying. Previous measurements of the water content in similar beads in the wet state gave the initial value [42]. The final value of the moisture content was approximated by water contents in dry paper sheets, usually around 4–6% depending on the type of paper and ambient moisture content [43].

A linear-elastic material model describes the constitutive behaviour of the underlying surface, with an elastic modulus >10 times the stiffness of the bead and the same Poisson’s ratio. Together with the fixed constraint applied at the bottom edge, the surface does not deform noticeably in relation to the deformation of the bead.

#### 2.2.3. Contact Description

The contact between the bead and the surface is defined by a surface pair with a node-to-surface formulation. As the softer body, the bead is the secondary surface, while the underlying body is the primary surface.

**Figure 4:**
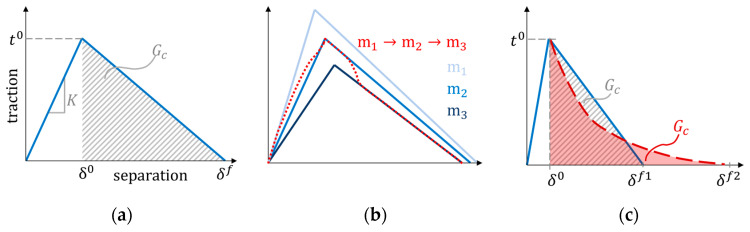
Schematic of (**a**) traction–separation law, (**b**) contact constitutive behaviour under moisture change during loading, and (**c**) example of cohesive law with similar fracture energy and damage initiation tractions but different softening behaviours.

The adhesion in the contact zone is modelled by a cohesive interaction. It is similar to the implementation of cohesive elements [44], but the surface has no volume. The cohesive behaviour describes the loads that arise when the surfaces attempt to separate. Separation can occur in two directions: normal and shear. The normal direction only considers tensile separation, as compressive stresses only push the surfaces closer together. A traction-separation law describes the relationship between the separation of the surfaces and the resulting tractions. This case uses a bilinear law consisting of three unique parameters, as seen in Figure 4a.

Here, the fracture energy $G_{c}$ and the damage initiation traction defined the cohesive degradation. Cohesive displacements in one direction do not cause cohesive stresses in the other, making the cohesive stiffness matrix uncoupled. Initially, the bilinear traction separation law defines the cohesive traction $t_{i}$ (i = n,s, either normal or shear mode) as dependent on the cohesive stiffness $K_{i}$ and the separation $\delta_{i}$

(11) $$t_{i}=K_{i}\delta_{i},$$

until it reaches the damage initiation traction $t_{i}^{0}$

(12) $$\max\left(\frac{t_{n}}{t_{n}^{0}},\frac{t_{s}}{t_{s}^{0}}\right)=1,$$

where $t_{n}$ and $t_{s}$, are the tractions in the normal and shear directions. The brackets  ⋅  are the Macauley operator, only accounting for positive values (damage does not evolve in compression). After damage initiation, the traction drops until it reaches the failure separation $\delta^{f}$, or releases the fracture energy $G_{c}$. The damage parameter D degrades the tractions after passing the peak as

(13) $$\begin{array}{c} t_{n}=\{\begin{array}{c} \left(1-D\right){\bar{t}}_{n}\;\;\;\;,\;\left({\bar{t}}_{n}\geq 0\right),\\ {\bar{t}}_{n}\;\;\;\;\;\;\;\;\;\;\;\;\;\;\;\;\;\;\;,\;\left({\bar{t}}_{n}<0\right), \end{array}\\ t_{s}=\left(1-D\right){\bar{t}}_{s}, \end{array}$$

where the nominal tractions ${\bar{t}}_{i}$ are tractions calculated from the nominal cohesive stiffness, see Equation (11). An effective measure accounts for the contributions from the normal and shear directions

(14) $${\left(\cdot \right)}_{m}=\sqrt{{\left(\cdot \right)}_{n}{}^{2}+{\left(\cdot \right)}_{s}^{2}},$$

where (⋅) is the relevant property. The damage parameter depends on the effective displacement,

(15) $$D=\frac{\delta_{m}^{f}\left(\delta_{m}^{max}-\delta_{m}^{0}\right)}{\delta_{m}^{max}\left(\delta_{m}^{f}-\delta_{m}^{0}\right)}.$$

where $\delta_{m}^{max}$ is the maximum value of the effective displacement $\delta_{m}$ to have occurred (D can not decrease) and $\delta_{m}^{0}$ is the damage initiation value. The fracture energy and effective traction at damage initiation $t_{m}^{0}$ (defined according to Equation (14)) define the effective displacement at failure

(16) $$\delta_{m}^{f}=2G_{c}/t_{m}^{0}.$$

The cohesive model includes moisture dependence by prescribing different parameters for different moisture levels, in between which the parameters vary linearly, as illustrated in Figure 4b. The damage evolution process (post-peak behaviour) can have a different softening behaviour than linear, see Figure 4c, but previous studies show that this has little influence on the results for loading in tension unless the adhesive material is highly ductile [45,46]. The parameter study considers this by varying the softening law, see Section 2.4.

#### 2.2.4. Numerical Aspects

Examining the energy outputs in the model is a way to ensure the credibility of the modelling procedures. The energy outputs were all positive, meaning that the model dissipates and does not generate energy. Running the simulations with a halved element size (in the finest region at the contact zone) assured a mesh-independent solution. It gave the same result in terms of contact stresses and deformation. However, changing the element size introduced convergence issues for the solution. Since the elements in the bead shrink significantly while the underlying surface does not shrink at all, it is believed that a size mismatch in the contact algorithm causes the problems. Moreover, this made the solution sensitive to parameter variations. Using other parameters or mesh would require a different mesh strategy, such as an adaptive procedure. Unlike the current implementation, an adaptive procedure allows node positions to adjust to the deformation thus preventing excessive distortion and size mismatch.

### 2.3. Experimental Comparison

**Figure 5:**
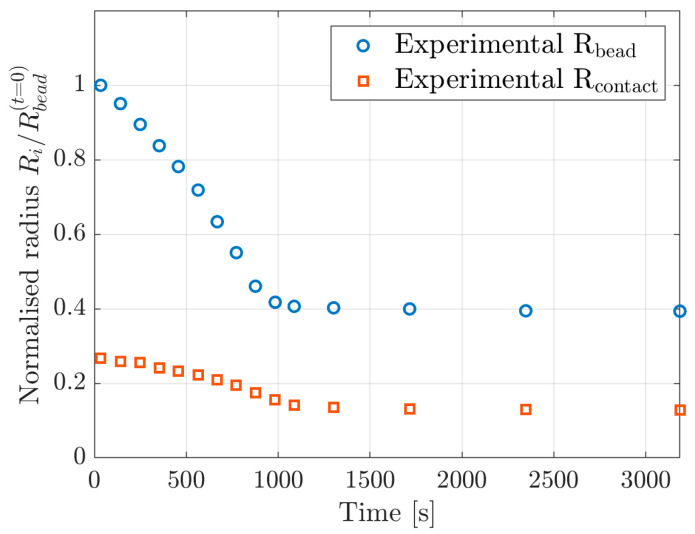
Drying geometry of water-swollen cellulose beads replotted from Li et al. [15]. The radii are normalised against the initial radius of the bead.

A trial-and-error procedure comparing the experimental geometry as measured by Li et al. [15] to the simulation results resulted in the material parameters summarised in Table 1. Figure 5 shows the experimental data regarding the radius of the bead and the radius of the contact area between the bead and the underlying surface.

### 2.4. Parameter Study

A parameter study followed the calibration of the fitting parameters. Introducing variations to the model parameters showed their influence on the overall response. The influence is summarised by observing the effect on the resulting bead geometry, and by comparing the stresses at select locations in the model for the different variations.

**Figure 6:**
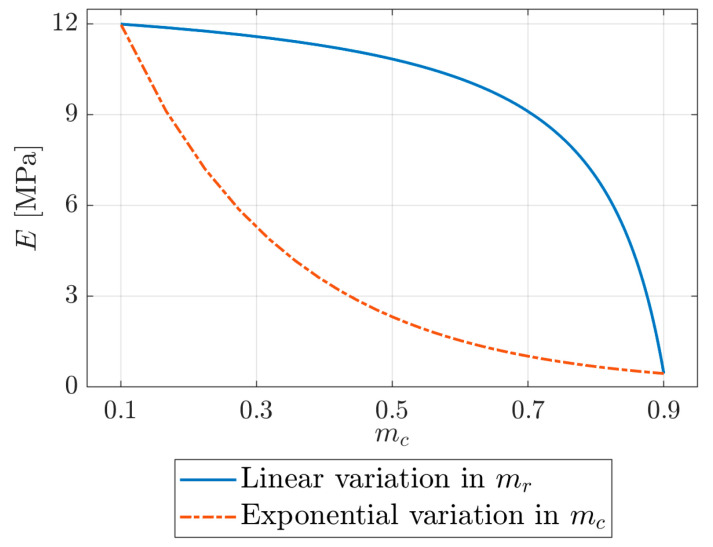
Development of the elastic modulus E with moisture content $m_{c}$ for the two tested cases.

The first step in the parameter investigation examined the effect of changing the constitutive behaviour of the bulk material by altering the evolution of the elastic modulus with moisture, so that the change was slow at first and then accelerated by the end. An exponential variation in $m_{c}$ replaced the linear variation in $m_{r}$, see Figure 6. The hyperelastic parameters were determined with the same procedure as before, using the resulting elastic modulus, as previously described in Section 2.2.2.

The second part of the parameter study examined the effect of changing the cohesive law. Changing the softening behaviour, while keeping the fitting parameters constant, allowed for studying its impact on the solution (see Figure 4c for an example of different softening behaviours). Replacing the linear softening as defined in Equation (15) with an exponential gives the damage variable as

(17) $$D=\int_{\delta_{m}^{0}}^{\delta_{m}^{f}}\frac{t_{m}}{G_{c}-G_{0}}d\delta ,$$

where $G_{0}$ is the elastic energy at the onset of damage.

## 3. Results

### 3.1. Parameter Fitting to Experimental Measurements

**Figure 7:**
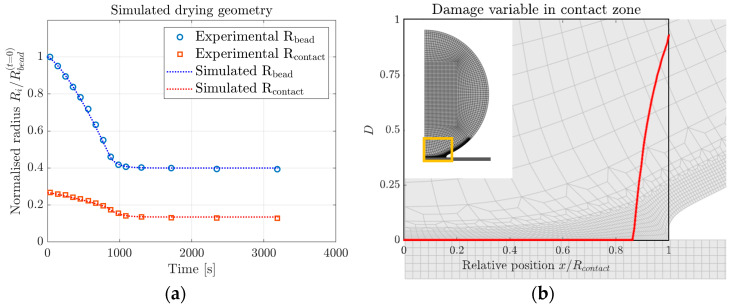
Results from simulations: (**a**) geometry evolution obtained by fitting of drying and diffusion coefficients, compared to experimental data replotted from Li et.al. [15] and (**b**) damage variable D (red) plotted for the last time step in the deformed model.

**Figure 8:**
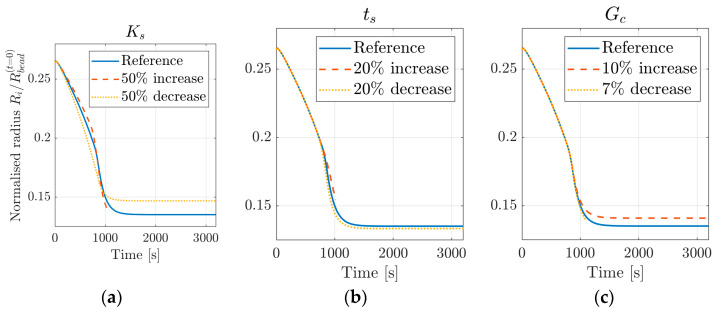
Parameter variations for (**a**) the cohesive stiffness in the shear direction $K_{s}$, (**b**) the cohesive strength in the shear direction $t_{s}^{0}$, and (**c**) the fracture energy $G_{c}$. The reference curves correspond to the values given in Table 2.

Figure 7a shows the simulated bead and contact radii resulting from the parameter fit, together with the experimental data. Figure 7b presents the resulting damage variable D, as defined in Equation (15), plotted against the deformed shape of the bead. Table 2 shows the determined fitting parameters. Figure 8 presents the resulting bead contact radius when varying some of the cohesive fitting parameters. The variation of remaining cohesive parameters (cohesive stiffness and strength in the normal direction, $K_{n}$ and $t_{n}^{0}$, respectively) gave no difference in results when changing the parameters by up to 50%.

### 3.2. Effect of Parameter Variation

**Figure 9:**
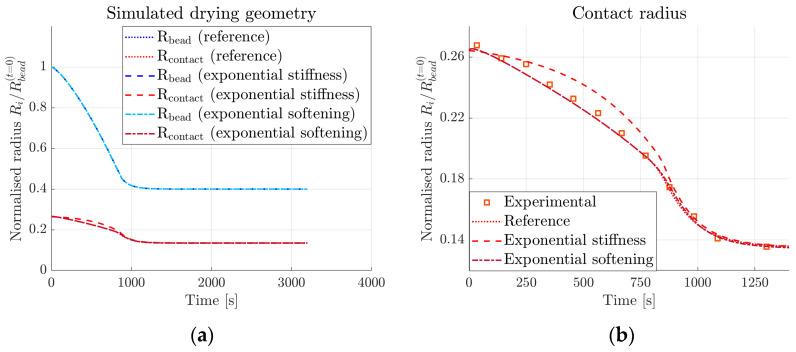
Simulation results for (**a**) bead and contact radii compared for reference simulations (as described in Section 3.1) and custom stiffness and exponential damage softening (as described in Section 2.4), and (**b**) a close-up of simulated contact radii compared to experimental data.

**Figure 10:**
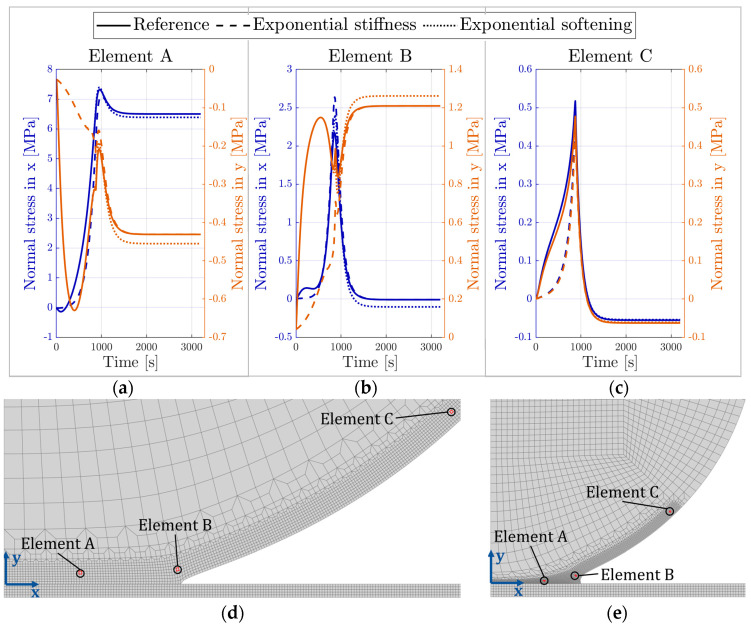
Element investigation: (**a**–**c**) stresses in two directions in three elements of the model for the parameter variations, and position of the elements in the model (**d**) before the onset of shrinkage and (**e**) after finalised shrinkage.

Figure 9 compares previously simulated reference curves (as shown in Figure 7a) to the resulting drying geometry with the customised development of the elastic modulus and exponential damage softening. In Figure 9a, both the bead and contact radii are shown for the different simulations. Figure 9b shows a close-up of the contact radius results and in addition, comparison to the experimental data. Figure 10 presents the normal stresses in the tangential (x-direction) and perpendicular (y-direction) directions for three different elements in the bead, placed slightly above the contact zone. Figure 10a–c compares the stress development over time in each element for the three different parameter sets (see Section 2.4). The positions of the elements are shown before the onset of shrinkage in Figure 10d and after finalised shrinkage in Figure 10e; the first element (from left to right) is the contact zone, the second is close to the edge of the contact zone, and the third one is outside the contact zone.

## 4. Discussion

The comparison between experimental measurements and simulated results (see Figure 7a) shows that the model is successful in replicating the observed drying geometry. The values presented for the fitting parameters in Table 2 show that varying all parameters linearly with moisture content is sufficient, suggesting that the model is well-suited for the application. Examining the damage value after the completion of drying, as shown in Figure 7b, shows that the edge of the contact zone is close to complete degradation, with a maximum value of D = 0.93.

The resulting material parameters, as presented in Table 2, give insight into what is happening with the material as it dries. The diffusion coefficient decreases with decreased moisture, while the expansion coefficient instead increases. As opposed to the onset of drying, the moisture gradient is higher in the late stages. Moreover, the shrinkage is also greater and thus, the developed stresses will be larger. Looking at the cohesive material parameters, the high tensile stiffness $K_{n}$ allows little to no separation between the contacting surfaces, while the lower value of $K_{s}$ allows some slippage in the shear direction. However, the shear stiffness increases over the course of drying, while the maximum shear traction $t_{s}^{0}$ and the fracture energy $G_{c}$ remain constant. Consequently, the joint becomes more brittle as it dries, which translates well to fibre networks; the material can be highly ductile in the wet state, but brittle when dry.

At closer examination, only contact stresses in the shear direction contribute to the degradation of the cohesive interaction. Consequently, the result is insensitive to higher values of cohesive damage initiation traction in the normal direction $t_{n}^{0}$. Another set of data would thus be necessary to determine the normal strength of the adhesive zone. The model could then be applied to pull-off measurements, where the force required to pull the bead off the surface is measured. The method developed here presents a good starting point for such an investigation, with the shear parameters already determined. Since the shear mode dominates this part of the analysis, further analysis should also investigate coupled effects, where the damage initiation and evolution depend on the normal and shear contributions nonuniformly.

As shown in Figure 8, the initial contact radius is insensitive to variations in cohesive parameters. However, several cohesive parameters affect the final contact radius. It raises the question of the uniqueness of the determined parameter set, which also requires further examination.

As expected, changing the stiffness evolution of the bead does not affect its shape during drying, as shown in Figure 9a. The hygroscopic strains are only dependent on the moisture expansion coefficient and the moisture content, see Equation (10). Changing the damage evolution behaviour also has no effect. However, changing the stiffness evolution does visibly affect the development of the contact radius, see Figure 9b. The lower stiffness in the initial stages (see Figure 6) will cause the cohesive tractions to be lower as well and damage will develop slower, therefore delaying decohesion (radius decrease). Since the final stiffness values are the same in both cases, the final values for the contact radius are the same, as the exponential variation increases the stiffness rapidly by the end. To understand how different stiffness values during drying impact the damage evolution in the normal direction, there needs to be further investigation. A possible course of action is, for example, studying pull-off measurements in intermediate states, prior to the complete drying of the bead. Subsequently, the experimental results can be compared to simulations with different stiffness evolution.

Changing the damage evolution shape has a negligible effect on the contact radius, as shown in Figure 9b. However, there is an effect on the residual stresses after completed drying. The curves in Figure 10a–c show that the peak stresses occur in all locations at almost the same time. The observed peak corresponds to the time when the moisture difference between the bulk of the bead and the ambient conditions stabilises. With one exception, the absolute value of the stresses decreases after this point, see Figure 10a–c. Presumably, the vanishing moisture gradient causes this drop. However, the stress in the y-direction close to the contact zone (element B) experiences an increase, see Figure 10b. A possible explanation is that the surrounding elements compress the edge of the contact zone in the presence of the moisture gradient, and when it disappears, the tensile stresses increase.

The changing of stiffness evolution introduces differences in the stresses compared to the reference model, which is apparent in all three examined elements. Close to the edge of the contact zone and outside of it, corresponding to element B and C (see Figure 10), respectively, the numerical values are similar, but the delayed stiffening also delays the stress development. Inside the contact zone the stress curves in the y-direction look initially very different. The slower development of stresses delays the development of damage. However, this difference in stress is not as large in the x-direction, where all three curves agree very well.

Analysing the effect of changing the softening behaviour from linear (as in the reference simulation) to exponential, proves that the effect on the stress evolution is small. However, there is a visible difference in the final stress value in the elements within the contact zone. The damage parameter D, as defined in Equations (15) and (17), will have different values in the two cases. The tractions are thus not equally reduced, causing the resulting residual stresses to differ. The difference is mostly small (1–6%), except in the case of the parallel stress close to the edge of the contact zone. The residual stress in the reference case is almost zero, but with the exponential damage evolution it results in a slight compressive stress.

Being able to examine what happens in the adhesive zone in detail makes the developed model useful for future evaluations of adhesion in model surfaces. As mentioned previously, pull-off tests will be of particular interest to further the understanding of other loading modes and adhesive failure. In a recent study Li et al. measured the adhesion between cellulose beads and a cellulose thin film [47]. In the pull-off test, the force required to separate surfaces is measured, but it says little about the stress state in the contact zone. The proposed model makes it possible to link global quantities, such as the pull-off force or drying geometry, to the stress state in the contact zone and degree of decohesion.

Another interesting application is studying the adhesion in the intermediate state, before complete drying, to understand how the moisture gradient affects the adhesion. As previously shown, mechanical properties and the internal structure change during drying [14,15,42], which is why it would be interesting to investigate the differences and similarities to changes in adhesive properties: the model can estimate cohesive properties and residual stresses. In addition to investigating the surface chemistry and how it contributes to the adhesion, the impact of drying can be explored, and the findings transferred to explain what happens when a fibre–fibre joint is formed.

For stronger interactions, e.g., when cellulose interacts with cellulose, the model could capture other phenomena important in contact creation. For example, in the case of a cellulose bead placed on a deformable film, wrinkling or other significant deformation of the film may occur. This model allows for studying this virtually, before doing complicated and time-consuming experiments. It is valuable both for designing and interpreting experiments.

However, the model might need further development to capture all major phenomena. For example, when the bead shrinks, the introduced deformations are very large. Because of the adhesive forces, the contact radius does not shrink proportionally to the bead radius. In cases with even larger adhesive forces, like cellulose–cellulose interactions, this mismatch will grow. Increased stresses close to the edge of the contact zone, greater strain gradients in the bead, and mesh distortions will follow, making the problem numerically more complex to solve. In this case, a different mesh approach would be suitable. The current approach uses the Lagrangian method, where the mesh nodes follow the deformation of the body; instead, an adaptive mesh could be introduced, where the distortion is limited.

Another aspect of the model that might need further development is the material assumptions. The hyperelastic model allowed for modelling soft behaviour with large deformations, although little is known about the constitutive behaviour of the beads. In particular, the material dependence on moisture and by extension, the moisture field in the bead during drying are interesting for future examination. By comparing the model to new experimental data, the material assumptions can be further evaluated and refined if needed.

Similarly, the description of the adhesive forces needs evaluation. The cellulose model surfaces intend to reflect simplified surfaces of fibres and capture interactions between them. However, which forces act in a fibre joint is still not completely determined. Molecular bonds, friction, and capillary forces are examples of physical phenomena believed to play a role in the fibre joint strength. Here, a cohesive description based on two principal directions, normal and tangential to the surface, with irreversible damage development replaces these. The model with the fitted parameters was fit to successfully replicate the drying geometry, but further investigation is needed to determine if the proposed description sufficiently replaces the adhesive forces.

To summarise, this paper presents a useful model for the investigation of adhesive forces between a bead and a surface. It is shown to be successful in replicating the observed drying geometry of a cellulose model bead and a rigid surface. Evaluation and comparison to pull-off experiments will further the understanding of the drying process in the formation of fibre–fibre joints. In particular, the model makes it possible to study an, at present, unmeasurable property: the development of stresses in the contact zone. The well-defined geometry of the cellulose bead model surface makes it possible to isolate adhesion from other phenomena and the findings can be implemented on more complex geometries, for example, fibre joints. The model also offers the possibility of implementation in fibre network models, with a more authentic description of the joint mechanics.

## 5. Conclusions

This paper presents a model for understanding the development of adhesion of cellulose beads during drying. The developed model is useful in furthering the knowledge of fibre joint formation in fibre network materials, and the most important conclusions are presented below.

- The developed model successfully replicates the drying geometry observed from experiments with parameters varying linearly with moisture.
- Several fitting parameters have similar effects on the solution; therefore, determination of a unique solution needs more examination.
- The validity of the model assumptions needs further investigation, especially in terms of moisture dependence and material parameters.
- It is a valuable tool for understanding the development of stresses during drying, and implementation in pull-off tests of model surfaces can further the insights.
- By determining the cohesive material parameters, it is shown that the shear mode completely dominates the decohesion during drying.

## Figures and Tables

**Table 1:** Material parameters in the model determined from experimental comparison.

Notation	Unit	Description
α	mm^2^/s	Diffusion coefficient (Equation (9)
β	-	Expansion coefficient (Equation (10))
$K_{n}$	MPa/mm	Cohesive stiffness in the normal direction (Equation (11))
$K_{s}$	MPa/mm	Cohesive stiffness in the shear direction (Equation (11))
$t_{n}^{0}$	MPa	Maximum cohesive traction in the normal direction (Equation (12))
$t_{s}^{0}$	MPa	Maximum cohesive traction in the shear direction (Equation (12))
$G_{c}$	N/mm	Cohesive fracture energy (Equation (16))

**Table 2:** Resulting material parameters in the model given for the initial and final values of the moisture content, $m_{c}^{0}$ and $m_{c}^{\infty }$, respectively.

Notation	Unit	Value	
		$m_{c}^{0\;}=0.9$	$m_{c}^{\infty }=0.1$
α	mm^2^/s	5.0 × 10^−4^	5.0 × 10^−5^
β	-	0.50	0.75
$K_{n}$	MPa/mm	8.0 × 10^6^	8.0 × 10^6^
$K_{s}$	MPa/mm	10	300
$t_{n}^{0}$	MPa	1.0 × 10^3^	1.0 × 10^3^
$t_{s}^{0}$	MPa	6.0	6.0
$G_{c}$	N/mm	0.27	0.27

## Data Availability

The experimental data used is found in the literature, simulated data can be acquired upon request from the corresponding author.
